# Abnormal coherence and sleep composition in children with Angelman syndrome: a retrospective EEG study

**DOI:** 10.1186/s13229-018-0214-8

**Published:** 2018-04-27

**Authors:** Hanna den Bakker, Michael S. Sidorov, Zheng Fan, David J. Lee, Lynne M. Bird, Catherine J. Chu, Benjamin D. Philpot

**Affiliations:** 1 Department of Cell Biology and Physiology, University of North Carolina, Chapel Hill, NC 27599 USA; 2 Carolina Institute for Developmental Disabilities, University of North Carolina, Chapel Hill, NC 27599 USA; 3 Neuroscience Center, University of North Carolina, Chapel Hill, NC 27599 USA; 4 Department of Neurology, University of North Carolina, Chapel Hill, NC 27599 USA; 5 Department of Neurosciences, University of California, San Diego, CA USA; 6 Department of Pediatrics, University of California, San Diego, CA USA; 7 Division of Dysmorphology/Genetics, Rady Children’s Hospital, San Diego, CA USA; 8 Department of Neurology, Massachusetts General Hospital, Boston, MA 02114 USA; 9 Harvard Medical School, Boston, MA 02215 USA

**Keywords:** Angelman syndrome, UBE3A, EEG, Coherence, Spindles, Biomarker

## Abstract

**Background:**

Angelman syndrome (AS) is a neurodevelopmental disorder characterized by intellectual disability, speech and motor impairments, epilepsy, abnormal sleep, and phenotypic overlap with autism. Individuals with AS display characteristic EEG patterns including high-amplitude rhythmic delta waves. Here, we sought to quantitatively explore EEG architecture in AS beyond known spectral power phenotypes. We were motivated by studies of functional connectivity and sleep spindles in autism to study these EEG readouts in children with AS.

**Methods:**

We analyzed retrospective wake and sleep EEGs from children with AS (age 4–11) and age-matched neurotypical controls. We assessed long-range and short-range functional connectivity by measuring coherence across multiple frequencies during wake and sleep. We quantified sleep spindles using automated and manual approaches.

**Results:**

During wakefulness, children with AS showed enhanced long-range EEG coherence across a wide range of frequencies. During sleep, children with AS showed increased long-range EEG coherence specifically in the gamma band. EEGs from children with AS contained fewer sleep spindles, and these spindles were shorter in duration than their neurotypical counterparts.

**Conclusions:**

We demonstrate two quantitative readouts of dysregulated sleep composition in children with AS—gamma coherence and spindles—and describe how functional connectivity patterns may be disrupted during wakefulness. Quantitative EEG phenotypes have potential as biomarkers and readouts of target engagement for future clinical trials and provide clues into how neural circuits are dysregulated in children with AS.

**Electronic supplementary material:**

The online version of this article (10.1186/s13229-018-0214-8) contains supplementary material, which is available to authorized users.

## Background

Angelman syndrome (AS) is a neurodevelopmental disorder caused by loss of neuronal expression of the maternally inherited *UBE3A* gene. Symptoms of AS include severe intellectual disability, impaired speech and motor function, epilepsy, sleep abnormalities, and some phenotypic overlap with autism [1–3]. Consistent and widespread electroencephalographic (EEG) irregularities in AS include epileptiform discharges, intermittent theta waves, and enhanced rhythmic delta waves [4–7]. In a prior study, we established that quantitative methods can be successfully applied to retrospective EEG data to confirm prior clinical descriptions of rhythmic delta in AS [6]. Here, we sought to use quantitative approaches to identify novel EEG signatures in the same groups of retrospective EEG data. We assessed EEG coherence during wakefulness and non-rapid eye movement (NREM) sleep and quantified sleep spindles during NREM sleep.

Coherence is a measure of how two simultaneously recorded EEG signals are correlated and represents a non-invasive approach to assess functional connectivity between brain areas [8]. We were motivated to study coherence in AS by the observation that individuals with autism show altered coherence patterns [9–17]. Autism has been recognized as a component feature of AS [18–22], and copy number increases in the 15q11-13 chromosomal region including *UBE3A* are also associated with syndromic autism [23, 24]. Some estimates suggest that up to ~ 50–80% of individuals with AS meet diagnostic criteria for autism [18]; however, these estimates vary greatly due to the difficulties assessing autism with standardized clinical tests in AS individuals. Traditionally, individuals with autism were thought to have comparatively high coherence between nearby electrode pairs (local hyperconnectivity) and low coherence between long-distance signals (global hypoconnectivity) [9–13], but this view has been challenged and become more nuanced in recent years [14–17, 25]. Thus, although specific connectivity patterns remain unclear, there is widespread consensus that EEG coherence is altered in autism. The phenotypic and genetic links between AS and autism led us to hypothesize that children with AS might also display irregularities in the relationship between long-range and short-range coherence.

Sleep abnormalities are common in individuals with AS [1–3, 26–34] and have also been reported in mouse models of the disorder [35, 36]. Sleep dysfunction includes arousal during sleep and short sleep duration, and has a major impact on the quality of life of individuals with AS and their caretakers [28–31]. We sought to identify quantitative EEG signatures underlying disrupted sleep patterns in children with AS. In addition to measuring coherence during sleep, we also quantified sleep spindles. Spindles are thalamocortical oscillations in the sigma band (~ 11–16 Hz) that occur during NREM sleep and are important for memory consolidation [37, 38]. Sleep spindle activity is decreased in a number of neurodevelopmental and neurodegenerative disorders, such as autism, intellectual disability, epilepsy, Alzheimer’s disease, and schizophrenia [39–46]. Although there have not yet been reports of substantial impairments in sleep architecture in AS, we hypothesized that quantitative measures might reveal subtle impairments in spindles and in patterns of sleep coherence that might be otherwise difficult to detect manually in a clinical EEG review setting.

During wakefulness, we report increased long-range EEG coherence in children with AS. During sleep, we also find increased long-range coherence, but specifically in the gamma band. We also report that sleep spindles are less frequent and shorter in children with AS. Overall, these measures provide insights into circuit-level neurobiology in AS and may have value as biomarkers or measures of target engagement for future therapeutic interventions. As this study was exploratory in nature, future work is needed to confirm coherence and spindle dysregulation in additional cohorts and to link these EEG phenotypes with behavioral outcomes.

## Methods

### Study design

We analyzed retrospective clinical EEGs from children with a genetically confirmed diagnosis of AS and age-matched neurotypical controls. All EEG studies were performed with the approval of institutional review boards (IRBs) at Harvard Medical School and UC San Diego, and consent was given to participate. All EEG data were previously analyzed for spectral content in our prior study [6], which tested the pre-defined hypothesis that delta rhythms are increased in AS. Here, we conducted an exploratory study to identify novel EEG phenotypes that could be measured quantitatively.

### Data sources

EEG data from 28 children with AS (14 male, 14 female) were recorded between 2006 and 2014 at the San Diego site (Rady Children’s Hospital San Diego) of the AS Natural History Study (ClinicalTrials.gov identifier: NCT00296764). EEG data from 72 neurotypical (NT) children (42 male, 30 female) were recorded at Massachusetts General Hospital between February 1, 2002, and May 1, 2012. All children were aged 4–11 years at the time of EEG recordings (AS 5.8 ± 0.3 years, NT 7.0 ± 0.2 years). Children with AS received EEG recordings as part of the Natural History Study, and neurotypical children were referred to Mass. General for diagnostic EEG evaluation. Only children that were subsequently determined to be non-epileptic and with documented normal neurodevelopment were included for analysis. All EEG data were gathered using the standard clinical method. Subjects were described in greater detail in a prior study [6], including AS molecular diagnosis, seizure history, and medications at the time of recording. An experienced clinical neurophysiologist assessed sleep/wake state in all recordings and categorized data into epochs of clear wakefulness and clear NREM sleep. The following signatures were used to identify NREM sleep: the presence of spindles, vertex waves, K-complexes, the absence of eye blinks, and/or decreased myogenic artifacts. Periods of REM sleep and periods where state was unclear were excluded. Subsequently, we had two separate datasets for analysis: one containing EEG signals during wakefulness (NT: *n* = 54; AS: *n* = 26), and one consisting of periods of NREM sleep (NT: *n* = 54; AS: *n* = 13).

### Data acquisition and pre-processing

EEGs were acquired using sampling rates ranging between 200 and 512 Hz using either Bio-Logic or Xltek systems and with standard 10–20 electrode placement. We pre-processed all data prior to this study using methods described in Sidorov et al. [6]. NT and AS EEGs were pre-processed in parallel using identical methods to limit the inherent impact of comparing recordings across two sites. Briefly, pre-processing consisted of re-referencing signals to linked ears, filtering, sleep/wake coding, and artifact removal. We used a second-order Butterworth filter with a high pass of 1 Hz, a low pass of 100 Hz, and a notch at 60 Hz (roll-off 40 dB/decade, attenuation −  0.263 dB at 2 Hz).

### Coherence analysis

We made group coherence comparisons (AS versus NT) separately in periods of wakefulness (Fig. 2, Additional file 1: Figure S1) and periods of NREM sleep (Fig. 3, Additional file 2: Figure S2). Within each EEG recording, we calculated the coherence between each of 145 electrode pairs in each of 100 frequency bins (1–50 Hz, 0.5 Hz bin size) by using the “mscohere” function in MATLAB [47–49]. The mscohere function calculates the magnitude-squared coherence, or how well “*x*” corresponds to “*y*” at each frequency, for each window (we used 2-s windows with 50% overlap), and averages these windows using Welch’s averaged, modified periodogram method. The coherence value of signals *x* and *y*, *C*_*xy*_(*f*), was calculated as a function of the spectral densities of signal *x*, *P*_*xx*_(*f*), and *y*, *P*_*yy*_(*f*), and the cross spectral density of *x* and *y*, *P*_*xy*_(*f*):

$$ {\boldsymbol{C}}_{\boldsymbol{xy}}\left(\boldsymbol{f}\right)=\frac{{\left|{\boldsymbol{P}}_{\boldsymbol{xy}}\left(\boldsymbol{f}\right)\right|}^{\mathbf{2}}}{{\boldsymbol{P}}_{\boldsymbol{xx}}\left(\boldsymbol{f}\right){\boldsymbol{P}}_{\boldsymbol{yy}}\left(\boldsymbol{f}\right)} $$

Pre-processed EEG signals were non-continuous due to sleep/wake coding and artifact removal. Thus, we calculated coherence separately within each segment of pre-processed data, then averaged coherence from different segments while weighting segment length. We only included continuous data longer than 10 s for coherence analyses.

To simplify interpretation of 14,500 coherence measurements per recording (145 electrode pairs × 100 frequency bins), we grouped data in two ways: (1) by frequency and (2) by electrode location.

#### Frequency grouping

To assess coherence within frequency bands of interest, we grouped and defined delta as 2–4 Hz, theta as 4–7 Hz, alpha as 8–12 Hz, beta as 12–30 Hz, and gamma as 30–50 Hz (Fig. 2, Fig. 3). To ensure statistical normality, coherence values (*R*^*2*^) from each 0.5 Hz frequency bin were first z-transformed using Fisher’s *r* to *z*. Then, the *z*-scores were averaged within each of the five frequency bands of interest. These averaged scores were then back-transformed using the Fisher inverse function, to obtain one *z*’-coherence value per electrode pair per frequency band [48–51].

#### Location grouping

**Fig. 1:**
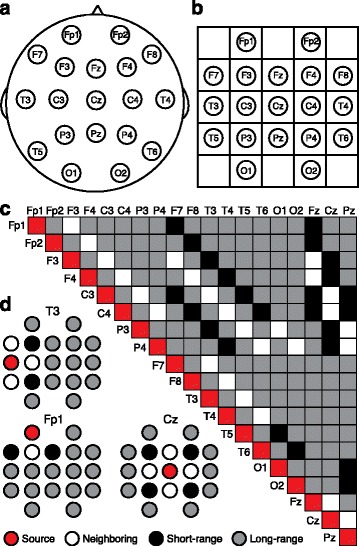
Defining long-range and short-range electrode pairs for coherence analyses. Standard 10–20 EEG electrode placements **a** on the scalp and **b** on a grid. **c** Grouping of all electrode pairs into short-range (black) and long-range (gray). Neighboring electrode pairs (white) were excluded from analysis. **d** Three examples of source electrodes (red) and their relationships with all other electrodes

We grouped electrode pairs according to their distance from each other (short-range and long-range) and their position relative to each other (intra-hemispheric and inter-hemispheric). To group electrode pairs by distance, we arranged electrodes (Fig. 1a) into a grid (Fig. 1b), assigned coordinates to each electrode (e.g., (2,1) for F7), and calculated the Euclidean distance between all coordinate pairs using the MATLAB function pdist [52]. The squared Euclidean distance (*d*^2^) for coordinates “a” and “b” was calculated with the following equation, where *x*_*a*_ is the x-coordinate of “*a*” and *y*_*a*_ is the *y*-coordinate of “*a*”:

$$ {d}_{ab}^2={\left({x}_a-{x}_b\right)}^2+{\left({y}_a-{y}_b\right)}^2 $$

**Fig. 2:**
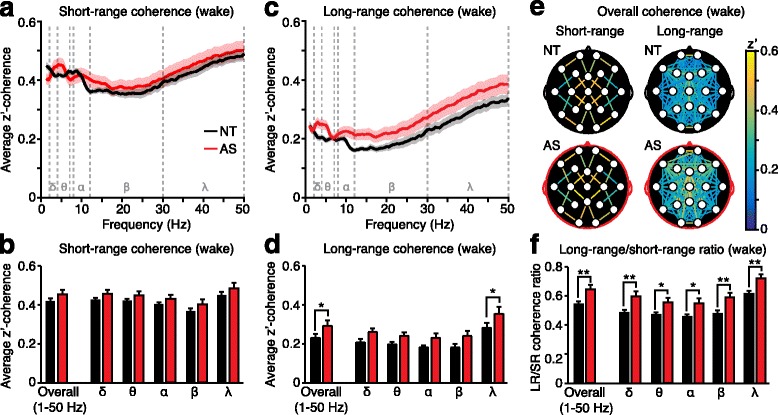
Long-range coherence during wakefulness is increased in AS. **a** Average short-range coherence across all frequency bands (delta *δ*, theta *θ*, alpha *α*, beta *β*, gamma *γ*). **b** Short-range coherence analyses grouped across all frequencies (“overall”) and by frequency. **c** Average long-range coherence across all frequency bands. **d** Long-range coherence analyses grouped overall and by frequency band. **e** Topographic coherence maps illustrating overall coherence between each short-range and long-range electrode pair on the surface of the skull. **f** Long-range coherence was broadly increased relative to short-range coherence within AS individuals. NT (black): *n* = 54, AS (red): *n* = 26

**Fig. 3:**
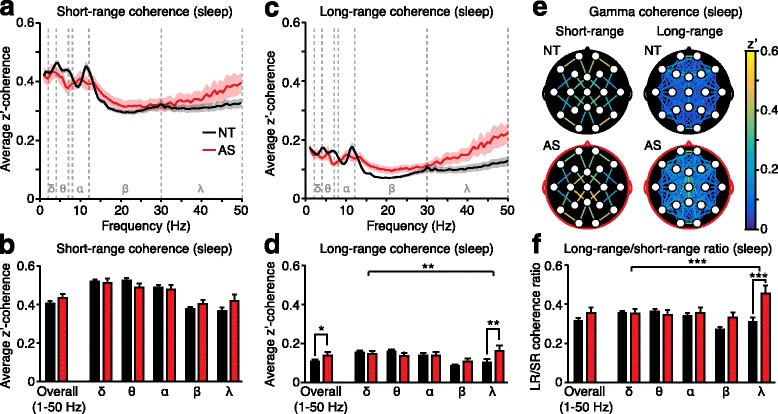
Long-range gamma-band coherence during sleep is increased in AS. **a** Average short-range coherence across all frequency bands (delta *δ*, theta *θ*, alpha *α*, beta *β*, gamma *γ*). **b** Short-range coherence analyses grouped across all frequencies (“overall”) and by frequency. **c** Average long-range coherence across all frequency bands. **d** Long-range coherence analyses grouped overall and by frequency band. **e** Topographic maps illustrate gamma coherence. **f** Long-range coherence was increased relative to short-range coherence specifically in the gamma band within AS individuals. NT (black): *n* = 53, AS (red): *n* = 12

Based on the Euclidean distance, we divided the electrode pairs into short-range pairs (*d*^2^ = 2) and long-range pairs (*d*^2^ > 2) (Fig. 1c–d). Directly neighboring electrodes (*d*^2^ = 1) were removed from analysis due to the potential confound of volume conduction [52]. We averaged *z*’-coherence values across all short-range electrode pairs (*n* = 24) and all long-range electrode pairs (*n* = 121) within each of the five frequency bands and overall (from 1 to 50 Hz) (Fig. 2, Fig. 3). When comparing intra-hemispheric coherence and inter-hemispheric coherence (Additional file 1: Figure S1B-G, Additional file 2: Figure S2B-G), we restricted intra-hemispheric analyses to long-range electrode pairs because by definition, all inter-hemispheric pairs were long-range. This approach eliminated the potential confound of short-range pairs in intra- but not inter-hemispheric data. We also excluded all pairs containing one or more midline electrode (Fz, Cz, Pz) from intra-versus-inter-hemispheric analysis. To graphically represent the spatial distribution of coherence, we created topographic coherence maps (Figs. 2e and 3e, Additional file 1: Figure S1D, S1G, Additional file 2: Figure S2A, S2D, S2G). These maps overlay the 10–20 system of electrode placement with color-coded lines indicating coherence between each electrode pair (averaged across all subjects).

To further evaluate the spatial profile of coherence phenotypes in AS, we calculated the coherence through individual nodes (electrodes) and through groups of nodes (Additional file 1: Figure S1H–I, Additional file 2: Figure S2H–I). First, for each electrode, we averaged coherence values for all long-range connections. Next, we averaged these individual-electrode averages for each spatially defined group of electrodes (frontal: Fp1, Fp2, F3, F4, F7, F8, Fz; central: C3, C4, Cz; temporal: T4, T5, T6; parietal: P3, P4, Pz; occipital: O1, O2).

### High-frequency artifact identification and removal

We entered coherence analyses with no pre-defined hypothesis regarding coherence in specific frequency bands. This unbiased approach revealed that children with AS showed increased long-range coherence in the gamma band (Fig. 3). However, accurately assessing gamma coherence is complicated by the possibility of electromyogenic (EMG) contamination of temporal signals in this bandwidth [53, 54]. Therefore, in addition to manual artifact removal at the initial stage of data pre-processing, we also conducted a post hoc analysis designed to identify low-amplitude EMG artifacts in sleep EEG data that are difficult to identify visually. Spectral power typically follows a ~ 1/f decay [55]; therefore, we excluded outliers in which the slope of the linear fit of the log power versus frequency (between 30–50 and 65–95 Hz) relationship in temporal electrodes exceeded − 1 [56]. We excluded one AS outlier and one NT outlier, in which muscle artifact likely corrupted interpretation of high-frequency coherence. We restricted these post hoc analyses to sleep EEGs, as altered coherence in wakeful EEGs was not specific to the gamma band and therefore not likely affected by high-frequency EMG artifacts.

### Consideration of volume conduction

We removed neighboring electrodes from analysis to minimize the effects of volume conduction [52]. To further assess the possible effects of volume conduction on the remaining electrode pairs, we performed a cross-correlation analysis on each one-second bin of continuous EEG signals and removed all bins in which the maximum cross-correlation between electrodes occurred at zero lag (Additional file 3: Figure S3). The average of all other bins provides a measure of cross-correlation, while robustly and conservatively accounting for the effects of volume conduction [57]. Generally, cross-correlation and coherence measures are expected to result in statistically similar findings [58]. We band-pass-filtered wake data (1–50 Hz) and sleep data (30–50 Hz) prior to cross-correlation analyses and grouped long-range and short-range electrode pairs.

### Spectral analysis

**Fig. 4:**
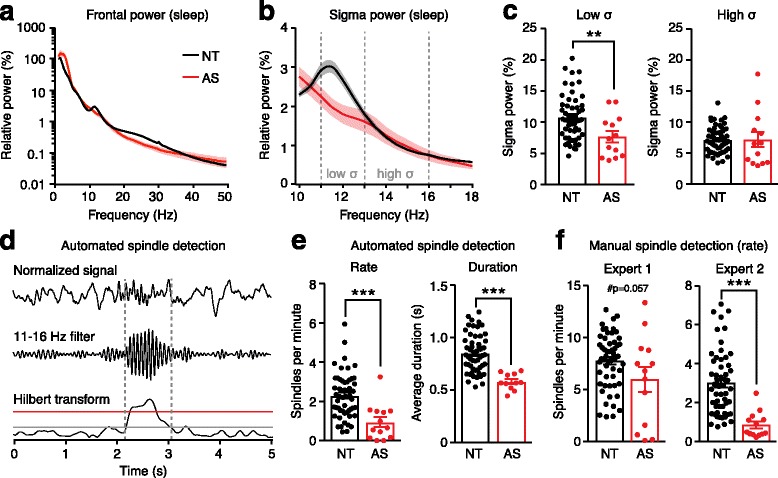
Sleep spindles are reduced in children with AS. Power spectra from frontal electrodes **a** across all frequencies from 1 to 50 Hz and **b** focused on the sigma bandwidth. Data were re-analyzed from Sidorov et al. [6]. **c** Children with AS showed decreased spectral power in the low sigma (11–13 Hz) band in which sleep spindles occur. **d** Steps in automated spindle detection: the normalized signal (top) is filtered (middle) and Hilbert-transformed to calculate instantaneous amplitude (bottom). The upper threshold (red) was used to detect spindles, and the lower threshold (gray) was used to define spindle duration. **e** Automated detection—spindle rate (NT: *n* = 54, AS: *n* = 13) and duration (NT: *n* = 54, AS: *n* = 11) were decreased in children with AS. **f** Manual detection—spindle rates as detected manually by two experts who were blinded to genotype

We re-analyzed and re-plotted the spectral power of frontal signals during sleep (Fig. 4a–c) using methods identical to our prior study [6], with one exception: here, we normalized power in each 0.5 Hz bin to the total power between 4 and 50 Hz, instead of to the total power between 1 and 50 Hz. We adjusted normalization to account for increased delta power (2–4 Hz) in children with AS. Thus, Fig. 4a represents the same data as Additional file 3: Figure S3J in Sidorov et al. [6].

### Spindle detection

We quantified the number and frequency of spindles during epochs of NREM sleep. We automated spindle detection using MATLAB using previously defined analysis parameters [59]. Automated spindle detection can be summarized in four steps (Fig. 4d): (1) To set the impedance levels of electrodes to similar levels, the detector normalized each pre-processed signal to the average power of the 90–100 Hz frequency range of that signal (Fig. 4d, top panel). (2) The data were filtered between 11 and 16 Hz using a 10th order Butterworth band-pass filter (Fig. 4d, middle panel). (3) The instantaneous amplitude was computed using a Hilbert transform and smoothed using a Gaussian kernel of 40 ms (Fig. 4d, bottom panel). (4) A spindle was detected if the instantaneous amplitude of the filtered signal crossed a threshold of 5.5 times the mean amplitude of the signal (red line in Fig. 4, bottom panel). When a spindle was detected, its duration was defined by when the signal crossed a lower threshold, 2.5 times the mean amplitude of the signal (gray line in Fig. 4d, bottom panel). Spindles were only counted if they were between 0.4 and 2.0 s in duration. Analyses of spindle frequency and spindle duration (Fig. 4e–f) represent total spindles across all 19 EEG channels. If two spindles were detected with an initiation interval of < 300 ms, these were considered to be a single event; thus, we did not double-count spindles seen at the same time across more than one channel. Two AS sleep EEGs had zero automatically detected spindles (Fig. 4e, left panel; *n* = 13); therefore, we excluded these recordings from analyses of spindle duration (Fig. 4e, right panel; *n* = 11).

Two trained clinical experts (DJL and ZF) manually analyzed spindle frequency in all sleep EEGs while blind to genotype (Fig. 4f). To ensure that experts remained blind, we filtered out background delta, which is highly prevalent in AS, with a 5 Hz high-pass filter prior to manual coding. Experts noted both the times at which spindles occurred and the confidence level of manually detecting spindles from background activity (high, medium, low).

### Statistical analyses

We used Student’s *t* tests to assess overall coherence (grouped across 1–50 Hz) as a function of genotype (Figs. 2b, d, f, and 3b, d, f, Additional file 1: Figure S1C, S1F, Additional file 2: S2C, S2F; “overall”). To assess the contribution of the five different frequency ranges (delta, theta, alpha, beta, gamma) to coherence, we used a two-way ANOVA with genotype and frequency as factors (Figs. 2b, d, f and 3b, d, f, Additional file 1: Figure S1C, S1F, Additional file 2: Figure S2C, S2F). We then used a post hoc test with Bonferroni’s correction for multiple comparisons to compare genotypes in individual frequency bands. We used Student’s *t* tests to assess cross-correlation, with volume conduction removed, as a function of genotype (Additional file 3: Fig. S3). We used Student’s *t* tests to compare spectral power, spindle frequency, and spindle duration between groups (Fig. 4c, e, f). We used two-tailed Fisher’s exact test to compare confidence in manual spindle detection. Cohen’s *d* effect sizes (Table 1) reflect overall (1–50 Hz) long-range/short-range coherence ratio (Fig. 2f, “overall”) during wakefulness, long-range/short-range gamma coherence ratio during sleep (Fig. 3f), spindle rate (Fig. 4e), and delta power averaged across all electrodes (re-analyzed from Sidorov et al. [6]). All statistical analyses were performed using GraphPad Prism 7. In all figures, the asterisk indicates *p* < 0.05, **p < 0.01, and ***p < 0.001. Where two-way ANOVAs were used, asterisks indicate statistically significant interactions (e.g., Fig. 3d, large brackets) and post hoc tests (e.g., Fig. 3d, gamma, small brackets). Main effects of genotype are noted in text. Error bars indicate SEM.

**Table 1:** Effect sizes of quantitative EEG phenotypes in children with AS. Altered coherence and decreased spindles are less robust than increased delta power

Measure	*p* value	Cohen’s *d*
Delta power (wake) [6]	< 0.0001	2.198
Overall coherence ratio (wake)	0.0016	0.747
Delta power (sleep) [6]	< 0.0001	2.058
Gamma coherence ratio (sleep)	< 0.0001	1.033
Spindle frequency (sleep)	0.0002	1.290

## Results

We calculated coherence between 145 combinations of 19 EEG electrodes for each individual and grouped coherence by short-range and long-range electrode pairs [52] (Fig. 1). To make group comparisons between children with AS and neurotypical (NT) children, we first assessed coherence across all frequency bands between 1 and 50 Hz (“overall coherence”) and then assessed coherence within frequency bands of interest (delta, theta, alpha, beta, gamma) while correcting for multiple comparisons, using Bonferroni’s multiple comparisons test. We analyzed EEG coherence separately in periods of wakefulness (NT: *n* = 54; AS: *n* = 26) and in periods of NREM sleep (NT: *n* = 54; AS: *n* = 13).

### Long-range coherence is increased in Angelman syndrome during wakefulness

During wakefulness, overall (1–50 Hz) short-range coherence (Fig. 2a) was not statistically different between children with AS and neurotypical controls (Fig. 2b, “overall”; *p* = 0.1887, Student’s *t* test). We next tested whether differences in short-range coherence would emerge within specific frequency bands. While two-way ANOVA revealed a statistically significant main effect of genotype (Fig. 2b; *F*_(1, 390)_ = 8.32, *p* = 0.0041), there was no genotype × frequency interaction (*F*_(4, 390)_ = 0.0702, *p* = 0.9910) and short-range coherence was not increased within any specific frequency band (post hoc Bonferroni tests: delta: *p* = 0.9113, theta: *p* > 0.9999, alpha: *p* > 0.9999, beta: *p* = 0.7041, gamma: *p* = 0.5514).

During wakefulness, overall (1–50 Hz) long-range coherence (Fig. 2c) was significantly increased in children with AS (Fig. 2d, “overall”; *p* = 0.0207). Two-way ANOVA revealed a significant main effect of genotype (Fig. 2d; *F*_(1,390)_ = 28.11, *p* < 0.0001) but no genotype × frequency interaction (*F*_(4,390)_ = 0.3385, *p* = 0.9224). While increased long-range coherence was detected statistically within the gamma band (post hoc tests: delta: *p* = 0.1258, theta: *p* = 0.3252, alpha: *p* = 0.1769, beta: *p* = 0.0559, gamma: *p* = 0.0105), the lack of genotype × frequency interaction indicates that this phenotype is not specific to any frequency band.

We next assessed whether increased long-range coherence in AS is expressed broadly across all electrode pairs or in a spatially restricted subset of connections or nodes. First, we created topographic coherence maps to visualize coherence in all electrode pairs (Fig. 2e). Comparison of NT and AS long-range maps suggests that increased long-range coherence is broadly spatially distributed. To quantify this comparison, we spatially grouped long-range electrode pairs: first, as a function of Euclidean distance, and next, by intra-hemispheric versus inter-hemispheric connectivity. Enhanced long-range coherence in AS was evident across a range of electrode distances (Additional file 1: Figure S1A), and in both intra-hemispheric and inter-hemispheric electrode pairs (Additional file 1: Figure S1B–G). We then asked if long-range coherence is selectively increased through specific nodes or groups of nodes. The lack of a significant genotype × region interaction effect demonstrated that increased long-range coherence in AS was not specific for individual electrodes or regions (Additional file 1: Figure S1H–I). Overall, we conclude that enhanced long-range coherence during wakefulness in AS is broadly distributed and is not specific to either certain groups of connections or certain groups of electrodes.

Coherence analyses grouped across individuals revealed that long-range coherence is increased in AS during wakefulness (Fig. 2c–d). Overall short-range coherence (grouped from 1 to 50 Hz) in AS individuals was statistically indistinguishable from NT individuals; therefore, we were surprised to find a significant main effect of genotype when including multiple comparisons across frequency bands (Fig. 2a–b). Thus, we next tested, within individuals, whether long-range coherence is meaningfully increased relative to short-range coherence. The ratio between long-range and short-range overall coherence (1–50 Hz) was increased in children with AS (Fig. 2f, “overall”; *p* = 0.0016). Two-way ANOVA revealed a significant main effect of genotype (*F*_(1,390)_ = 48.39, *p* < 0.0001), but no genotype × frequency interaction (*F*_(4,390)_ = 0.1083, *p* = 0.9796), and post hoc tests revealed that increased long-range to short-range coherence ratios were detectable in all frequency ranges tested (Fig. 2f; delta: *p* = 0.0037, theta: *p* = 0.0401, alpha: *p* = 0.0220, beta: *p* = 0.0040, gamma: *p* = 0.0063). Thus, we conclude that within individuals, long-range coherence is increased relative to short-range coherence in children with AS during wakefulness. Increased long-range coherence is evident across frequency bands.

### Long-range gamma-band coherence is increased in Angelman syndrome during sleep

During periods of sleep, overall (1–50 Hz) short-range coherence (Fig. 3a) was statistically comparable between AS and NT individuals (Fig. 3b, “overall”; *p* = 0.3059). Two-way ANOVA revealed no significant main effect of genotype (Fig. 3b; *F*_(1,315)_ = 0.002, *p* = 0.9672) and no interaction between genotype and frequency (Fig. 3b; *F*_(4,315)_ = 1.958, *p* = 0.1008). During sleep, overall long-range coherence (Fig. 3c) was increased in AS (Fig. 3d, “overall”; *p* = 0.0442). Increased long-range coherence was driven primarily by increased coherence in the gamma band (Fig. 3d; genotype × frequency interaction: *F*_(4,315)_ = 3.758, *p =* 0.0053; post hoc tests: delta, theta, alpha, beta: *p* > 0.75, gamma: *p* = 0.0024). Topographic coherence maps (Fig. 3e) and analysis (Additional file 2: Figure S2) suggest that increased long-range gamma coherence during sleep is broadly expressed (and not spatially restricted) in AS.

Within individuals, the ratio between long-range and short-range overall (1–50 Hz) coherence was not increased in children with AS (Fig. 3f, “overall”; *p* = 0.1824). Two-way ANOVA revealed a significant genotype × frequency interaction (*F*_(4,315)_ = 5.946, *p* = 0.0001), and post hoc tests revealed that there was an increase in coherence specific to the gamma band (Fig. 3f; delta, theta, alpha: *p* > 0.9999, beta: *p* = 0.1796, gamma: *p* < 0.0001). Gamma coherence is sensitive to electromyogenic (EMG) artifacts [53, 54]; therefore, we identified and excluded recordings in which these artifacts were present, yet were not manually excluded in the initial data pre-processing phase [56] (see the “Methods” section). These outliers (1 AS, 1 NT) have been excluded from Fig. 3, Additional file 2: Figure S2, and analyses. Overall, long-range coherence is increased in AS during sleep specifically in the gamma band.

### Coherence phenotypes in Angelman syndrome are not driven by group differences in volume conduction

Volume conduction of signals propagated from a common source may lead to identification of spuriously coupled scalp EEG signals. We tested whether volume conduction (instantaneous propagation of activity from sources to recording channels) was driving the coherence phenotypes in AS. We calculated cross-correlation and removed all periods where the maximum cross-correlation between electrode pairs occurred at zero lag. This approach is a robust and conservative way of removing potentially spurious electrode pairs [57]. With potential volume conduction excluded, genotype differences in long-range coherence persisted during both periods of wake and sleep (Additional file 3: Figure S3). With conservative removal of volume conduction, short-range gamma coherence was also statistically increased in AS EEGs during sleep. However, the long-range/short-range ratio remained elevated in AS, confirming that long-range coherence gamma coherence is elevated relative to short-range gamma coherence. Overall, differences in coherence between AS and NT groups are not the result of distortion due to volume conduction.

### Frequency and duration of sleep spindles is decreased in Angelman syndrome

Sleep spindles are visible in EEGs during NREM sleep as bursts of synchronous activity in the sigma band (11–16 Hz) [60]. In neurotypical children, we observed a local peak in sigma-band coherence during sleep (Fig. 3a, c) but not wakefulness (Fig. 2a, c) that may reflect the presence of sleep spindles [46, 61, 62]. We did not observe a sigma-band coherence peak in children with AS during sleep (Fig. 3a, c), suggesting that spindles may be decreased in AS. Spindle density also correlates with a peak in spectral power in the sigma band during NREM sleep [46]; therefore, we re-analyzed power spectra from our prior study [6] to focus on the sigma band during sleep. We confirmed that spectral power in the low sigma band (11–13 Hz) was decreased in children with AS (Fig. 4a–c; *p* = 0.0071). Together, decreased sigma coherence and spectral power during sleep provide indirect evidence suggesting that sleep spindles are dysregulated in AS.

We directly tested the hypothesis that sleep spindles are dysregulated in AS by using an automated spindle detection algorithm developed by Kim and colleagues [59] (Fig. 4d). Children with AS had fewer spindles (Fig. 4e; *p* = 0.0002), and the spindles were of shorter duration (Fig. 4e; *p* < 0.0001) than those of neurotypical controls. Although automation provides a fast and objective way to quantify sleep spindles, even established detection methods can be less accurate than human experts [63]. Therefore, we had two clinical experts manually count spindles in all sleep EEGs while blind to genotype. Results from expert 1 revealed a trend towards decreased spindle rate in children with AS (Fig. 4f; *p* = 0.0570). Results from expert 2 show a significant decrease in spindle rate in AS children (Fig. 4f; *p* < 0.0001). Expert 1 noted low confidence spindle detection for 11 of 13 AS EEGs and not for a single neurotypical EEG (*n* = 54; *p* < 0.0001, Fisher’s exact test). Expert 2 noted medium confidence for all recordings.

### Coherence and spindle dysregulation in AS have smaller effect sizes than delta power

Exploratory analyses of retrospective EEGs revealed coherence and spindle phenotypes in children with AS (Figs. 2, 3, and 4). In a prior study, we reported that children with AS also have increased delta power during both wakefulness and sleep [6]. Such quantitative EEG measures may have value as biomarkers or measures of target engagement for future clinical trials in AS. An important factor when considering biomarker viability is the reliability of a measure [64]. Therefore, we compared the Cohen’s *d* effect sizes for each quantitative EEG phenotype in AS (Table 1). Increased delta power was the most robust phenotype we assessed.

## Discussion

Quantitative EEG analyses revealed three phenotypes in children with AS that would otherwise be difficult to discern in a routine clinical or research setting: (1) increased long-range coherence during wakefulness, (2) increased long-range gamma-band coherence during sleep, and (3) decreased sleep spindle number and duration.

EEG coherence provides a measure of how neural activity is correlated between brain areas and is widely used as a proxy for functional connectivity [8]. Coherence measures the consistency of the phase and amplitude difference between EEG signals in a given frequency band. Coherence is thus distinct from spectral power, which measures the relative amplitude of electrical activity within a frequency band from a single electrode. Thus, despite robust increases in delta power [5, 6], children with AS have normal delta-band coherence (Figs. 2 and 3). While coherence and delta power phenotypes in AS are both ultimately caused by loss of neuronal UBE3A protein, they likely reflect different proximate circuit-level impairments.

During wakefulness, long-range EEG coherence was increased in children with AS across a broad range of frequencies (Fig. 2). Increased long-range coherence in AS was seen throughout the brain and was not driven by altered coherence in a spatially restricted subset of connections (Fig. 2e, Additional file 1: Figure S1). There is general consensus that functional connectivity is widely disrupted in autism [9–17, 25], and our findings confirm that coherence is also dysregulated in AS, a disorder with some autistic features. However, increased long-range functional connectivity may be surprising given prior studies of decreased structural connectivity in AS, both in mouse models [65] and patient populations [66, 67]. This suggests that despite reduced structural connectivity, there may be fewer inhibitory constraints on efferent projections in the AS brain.

During sleep, long-range coherence was significantly increased in children with AS, but only in the gamma band (Fig. 3). Gamma-band coherence is an indicator of attentive wakefulness [68], and accordingly, gamma coherence is typically lower during sleep than during wakefulness [69–71]. We confirmed that gamma coherence in neurotypical children is lower during sleep than during wake (compare Figs. 2 and 3). However, the pattern of elevated long-range gamma coherence during sleep in AS children resembles what is typically seen in a wakeful state. A common challenge in analyzing gamma-band coherence is the presence of electromyogenic artifacts, which are visible in EMG spectra and are often seen temporally in the gamma range [53, 54]. Therefore, we used an outlier analysis to exclude recordings in which EMG artifacts exceeded an established threshold [56]. Two additional pieces of evidence confirm that gamma coherence phenotypes in AS are not driven by EMG artifacts: (1) increased gamma coherence is specific to long-range electrode pairs and (2) gamma coherence is not increased specifically in temporal electrodes (Additional file 2: Figure S2I). Overall, long-range functional connectivity was increased in AS EEGs during both wake and sleep states. However, coherence patterns differed as function of state: phenotypes were gamma-specific during sleep and not frequency-specific during wake. Thus, it is critical to control for sleep state when assessing functional connectivity.

We also report that sleep spindles are shorter and less frequent in AS (Fig. 4). This finding is consistent with the decreased spindle frequency seen in autism, intellectual disability, and sleep disorders [39–43]. Despite many clinical studies of Angelman EEGs over the past 30 years, to our knowledge, there have been no reports to date of dysregulated spindles. This is surprising because unlike coherence, sleep spindles may be easily detected by the eye. However, subtle dysregulation of spindles may be difficult to gauge clinically, especially given the pervasive disruptions in background activity in AS [5]. Therefore, automated spindle detection using an unbiased, high-throughput method was used to determine that spindle rate and duration were decreased in AS EEGs. In addition, one of two blinded experts confirmed a statistically significant decrease in spindle rate in AS EEGs, with the other finding a strong trend. To enable blinded data analysis, we filtered out the delta activity that is pervasive in the AS EEG; however, this likely reduced both accuracy and confidence of manual detection. Future studies of sleep spindles in AS must consider and weigh the challenges of manual and automated detection, but we favor an automated approach because it is not subject to the reporter biases that plague qualitative outcome measurements in clinical trials.

More broadly, experimental conditions must be considered when evaluating our exploratory analyses of sleep composition in AS (both spindles and coherence). We used retrospective EEG data, which included periods of sleep and wake and was not designed explicitly as a sleep study. Because children with AS have pervasive sleep problems, it is likely that sleep quality during EEG recordings varied by group. For example, only 46% (13/28) children with AS slept during EEGs, whereas 75% (54/72) of neurotypical children slept. In addition, the nature of sleep during clinical EEG recordings may not be representative of typical overnight sleep. For example, the average length of NREM sleep during EEGs recordings was only ~ 14 min for neurotypical children and ~ 22 min for children with AS [6]. Thus we propose that sleep spindles and gamma coherence phenotypes should be explicitly tested in well-controlled overnight sleep studies.

Clinical trials are on the horizon for AS; therefore, development of biomarkers, outcome measures, and measures of target engagement are especially valuable. Biomarkers for AS need not have diagnostic value, as diagnoses are made genetically. Therefore major considerations in evaluating a biomarker include whether it is quantitative, easily measured, reliable, and linked to clinically meaningful outcomes [64]. Previously, we described enhanced delta rhythmicity in AS, which is quantitative, non-invasive, and reliable, but the link between delta rhythms and behavior has not yet been established. While effect sizes of gamma coherence and sleep spindle phenotypes are less than delta rhythms (Table 1), these phenotypes are likely linked to sleep quality. Therefore, they may be considered as biomarkers, particularly if a study is interested in quantifying sleep as a primary outcome measure. However, delta power is a substantially more robust biomarker, with only slight overlap between AS and neurotypical groups at the level of individuals. Future study of sleep biomarkers in an overnight setting, with AS and neurotypical children studied in parallel at a single site, may have the potential to decrease individual variability and increase robustness.

Quantitative EEG phenotypes may also provide insights into circuit-level biological mechanisms underlying AS. For example, mechanisms governing spindle initiation and propagation have been well characterized [37]. Spindles are driven by the intrinsic properties of, and interactions between, thalamocortical cells and thalamic reticular cells. Thalamocortical circuits, which also drive cortical delta rhythms [72], may be studied in mouse models to better understand how loss of UBE3A disrupts neural circuits. We hypothesize that loss of UBE3A from a small population of like neurons is sufficient to disrupt sleep spindles in AS. Coherence phenotypes, which are expressed broadly throughout the brain, are likely driven through different processes.

## Conclusions

Overall, we identified three novel quantitative EEG phenotypes in an exploratory analysis of retrospective EEGs from children with AS. These results have potential value as biomarkers and in pointing towards underlying neural substrates. Future work is needed to confirm findings in independent samples, particularly under conditions designed to study sleep explicitly.

## Additional files

Additional file 1: **Figure S1.** Spatial analysis of long-range coherence during wakefulness. (A) Overall coherence (1–50 Hz) during wakefulness as a function of Euclidean distance. Dotted line represents the cutoff between short-range and long-range coherence. Two-way ANOVA for long-range coherence: genotype: *F*_(1,774)_ = 40.53, *p* < 0.0001; distance: *F*_(9,774)_ = 22.75, *p* < 0.0001; interaction: *F*_(9,774)_ = 0.4326, *p* = 0.9187. (B) Raw and (C) grouped intra-hemispheric long-range coherence. Overall (1–50 Hz) intra-hemispheric coherence is increased in AS (*p* = 0.0145). Two-way ANOVA: genotype: *F*_(1,390)_ = 32.77, *p* < 0.0001; genotype × frequency interaction: *F*_(4,390)_ = 0.1419, *p* = 0.9665; post hoc tests: delta: *p* = 0.0646, theta: *p* = 0.1067, alpha: *p* = 0.1315, beta: *p* = 0.0521, gamma: *p* = 0.0078. (D) Topographic coherence maps for all intra-hemispheric electrode pairs. (E) Raw and (F) grouped inter-hemispheric long-range coherence. Overall (1–50 Hz) inter-hemispheric coherence was increased in AS (*p* = 0.0303). Two-way ANOVA: genotype: *F*_(1,390)_ = 22.49, *p* < 0.0001; genotype × frequency interaction: *F*_(4,390)_ = 0.3383, *p* = 0.8521; post hoc tests: delta: *p* = 0.2771, theta: *p* = 0.8276, alpha: *p* = 0.2657, beta: *p* = 0.0785, gamma: *p* = 0.0180. (G) Topographic coherence maps for all inter-hemispheric electrode pairs. (H) Overall (1–50 Hz) long-range coherence through individual electrodes and (I) electrodes grouped by region. Two-way ANOVA: genotype: *F*_(1,390)_ = 23.11, *p* < 0.0001; genotype × region interaction: *F*_(4,390)_ = 0.8003, *p* = 0.5255; post hoc tests: frontal: *p* = 0.0555, central: *p* = 0.0783, parietal: *p* = 0.0112, temporal: *p* > 0.9999, occipital: *p* = 0.2414. NT (black): *n* = 54, AS (red): *n* = 26. (PDF 271 kb)

Additional file 2: **Figure S2.** Spatial analysis of gamma-band coherence during sleep. (A) Gamma-band coherence during sleep as a function of Euclidean distance. Dotted line represents the dividing line between short-range and long-range coherence. Two-way ANOVA for long-range coherence: genotype: *F*_(1,629)_ = 30.93, *p* < 0.0001; distance: *F*_(9,629)_ = 15.46, *p* < 0.0001; interaction: *F*_(9,629)_ = 0.8704, *p* = 0.5516. Asterisk indicates significance by post hoc Bonferroni tests. (B) Raw and (C) grouped intra-hemispheric long-range gamma-band coherence. Overall: *p* = 0.0565; two-way ANOVA: genotype: *F*_(1,315)_ = 1.484, *p* = 0.2240; genotype × frequency interaction: *F*_(4,315)_ = 2.943, *p* = 0.0206; post hoc tests: delta, theta, alpha, beta: *p* > 0.9999, gamma: *p* = 0.0070. (D) Topographic coherence maps for all intra-hemispheric electrode pairs. LR long-range. (E) Raw and (F) grouped inter-hemispheric long-range coherence. Overall: *p* = 0.1139; two-way ANOVA: genotype: *F*_(1,315)_ = 0.409, *p* = 0.5230; genotype × frequency interaction: *F*_(4,315)_ = 3.303, *p* = 0.0114; post hoc tests: delta: *p* > 0.9999, theta: *p* = 0.4283, alpha, beta: *p* > 0.9999, gamma: *p* = 0.0140. (G) Topographic coherence maps for all inter-hemispheric electrode pairs. (H) Gamma coherence through individual electrodes and (I) electrodes grouped by region. Two-way ANOVA for region: genotype: *F*_(1,315)_ = 24.86, *p* < 0.0001; genotype × region interaction: *F*_(4,315)_ = 0.9112, *p* = 0.4576; post hoc tests: frontal: *p* = 0.3285, central: *p* = 0.0465, parietal: *p* = 0.0022, temporal: *p* > 0.9999, occipital: *p* = 0.1522. NT (black): *n* = 53, AS (red): *n* = 12. (PDF 503 kb)

Additional file 3: **Figure S3.** Coherence phenotypes persist with conservative exclusion of volume conduction. (A) Cross-correlation during wakefulness across all frequencies (1–50 Hz). Left panel: short-range electrode pairs (*p* = 0.0549). Center panel: long-range electrode pairs (*p* < 0.0001). Right panel: long-range/short-range ratio (*p* = 0.0027). (B) Cross-correlation during sleep in the gamma band (30–50 Hz). Left panel: short-range (*p* = 0.0004). Center panel: long-range (*p* < 0.0001). Right panel: long-range/short-range ratio (*p* = 0.0016). (PDF 405 kb)

## Abbreviations

AS: Angelman syndrome
EEG: Electroencephalography
EMG: Electromyography
NT: Neurotypical
NREM: Non-rapid eye movement sleep
