# Supplementary figures and images for: Abnormal coherence and sleep composition in children with Angelman syndrome: a retrospective EEG study
Source: Mol Autism. 2018 Apr 27;9:32. doi: 10.1186/s13229-018-0214-8 (PMC5924514; doi:10.1186/s13229-018-0214-8)

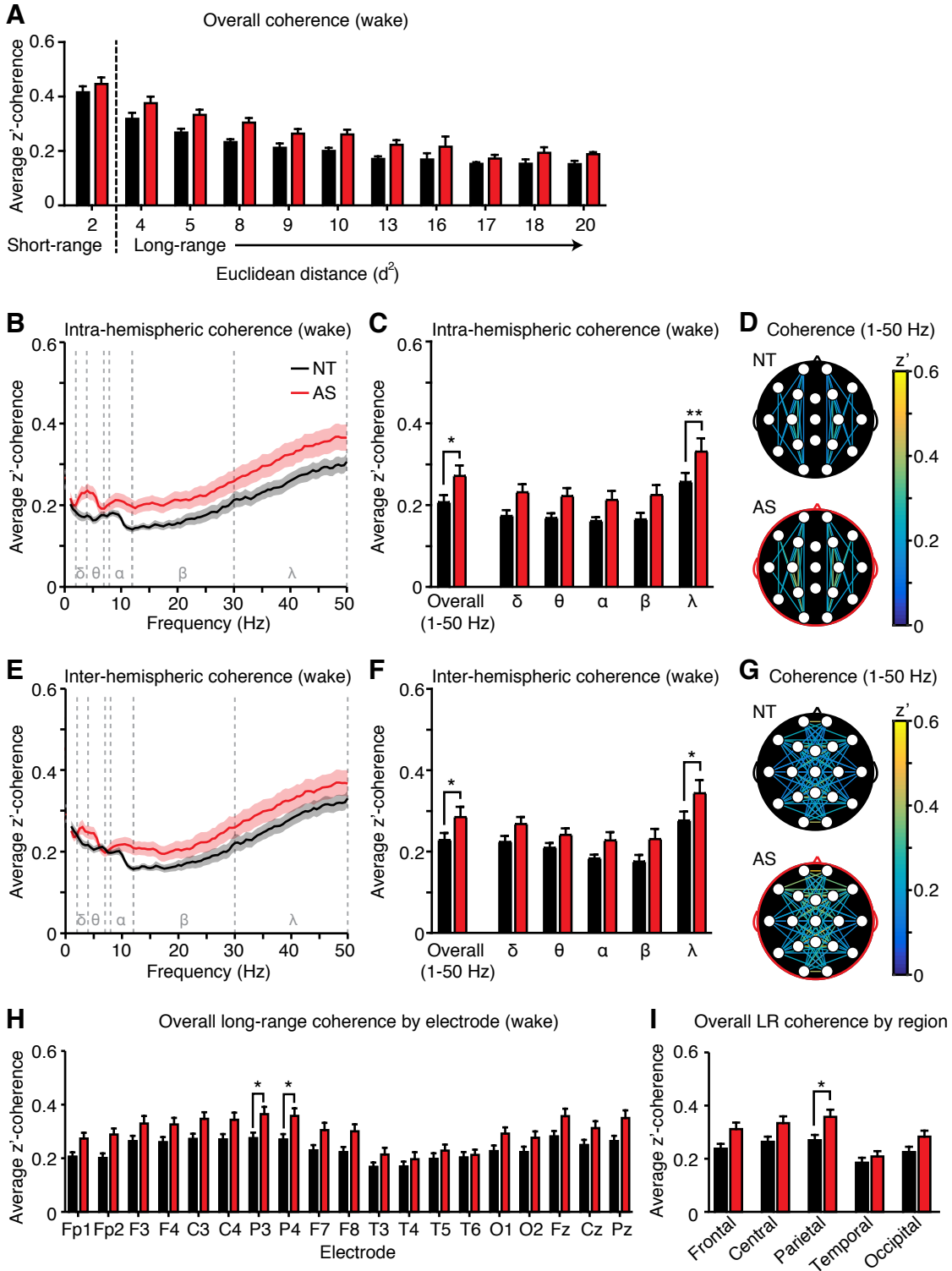

Supplement: Supplementary file 1 — Figure S1. Spatial analysis of long-range coherence during wakefulness. (A) Overall coherence (1–50 Hz) during wakefulness as a function of Euclidean distance. Dotted line represents the cutoff between short-range and long-range coherence. Two-way ANOVA for long-range coherence: genotype: F(1,774) = 40.53, p < 0.0001; distance: F(9,774) = 22.75, p < 0.0001; interaction: F(9,774) = 0.4326, p = 0.9187. (B) Raw and (C) grouped intra-hemispheric long-range coherence. Overall (1–50 Hz) intra-hemispheric coherence is increased in AS (p = 0.0145). Two-way ANOVA: genotype: F(1,390) = 32.77, p < 0.0001; genotype × frequency interaction: F(4,390) = 0.1419, p = 0.9665; post hoc tests: delta: p = 0.0646, theta: p = 0.1067, alpha: p = 0.1315, beta: p = 0.0521, gamma: p = 0.0078. (D) Topographic coherence maps for all intra-hemispheric electrode pairs. (E) Raw and (F) grouped inter-hemispheric long-range coherence. Overall (1–50 Hz) inter-hemispheric coherence was increased in AS (p = 0.0303). Two-way ANOVA: genotype: F(1,390) = 22.49, p < 0.0001; genotype × frequency interaction: F(4,390) = 0.3383, p = 0.8521; post hoc tests: delta: p = 0.2771, theta: p = 0.8276, alpha: p = 0.2657, beta: p = 0.0785, gamma: p = 0.0180. (G) Topographic coherence maps for all inter-hemispheric electrode pairs. (H) Overall (1–50 Hz) long-range coherence through individual electrodes and (I) electrodes grouped by region. Two-way ANOVA: genotype: F(1,390) = 23.11, p < 0.0001; genotype × region interaction: F(4,390) = 0.8003, p = 0.5255; post hoc tests: frontal: p = 0.0555, central: p = 0.0783, parietal: p = 0.0112, temporal: p > 0.9999, occipital: p = 0.2414. NT (black): n = 54, AS (red): n = 26. (PDF 271 kb) [file 13229_2018_214_MOESM1_ESM.pdf]

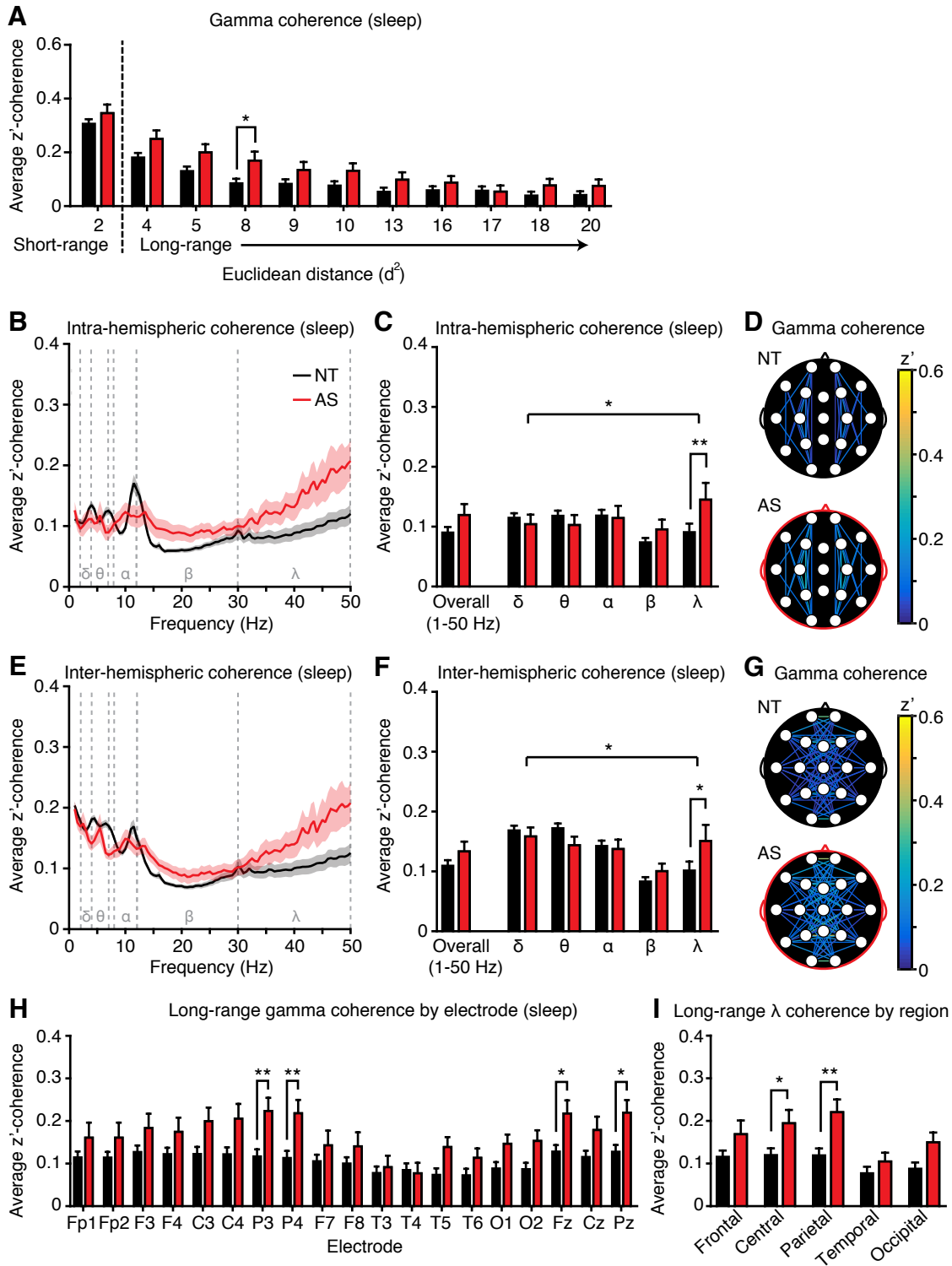

Supplement: Supplementary file 2 — Figure S2. Spatial analysis of gamma-band coherence during sleep. (A) Gamma-band coherence during sleep as a function of Euclidean distance. Dotted line represents the dividing line between short-range and long-range coherence. Two-way ANOVA for long-range coherence: genotype: F(1,629) = 30.93, p < 0.0001; distance: F(9,629) = 15.46, p < 0.0001; interaction: F(9,629) = 0.8704, p = 0.5516. Asterisk indicates significance by post hoc Bonferroni tests. (B) Raw and (C) grouped intra-hemispheric long-range gamma-band coherence. Overall: p = 0.0565; two-way ANOVA: genotype: F(1,315) = 1.484, p = 0.2240; genotype × frequency interaction: F(4,315) = 2.943, p = 0.0206; post hoc tests: delta, theta, alpha, beta: p > 0.9999, gamma: p = 0.0070. (D) Topographic coherence maps for all intra-hemispheric electrode pairs. LR long-range. (E) Raw and (F) grouped inter-hemispheric long-range coherence. Overall: p = 0.1139; two-way ANOVA: genotype: F(1,315) = 0.409, p = 0.5230; genotype × frequency interaction: F(4,315) = 3.303, p = 0.0114; post hoc tests: delta: p > 0.9999, theta: p = 0.4283, alpha, beta: p > 0.9999, gamma: p = 0.0140. (G) Topographic coherence maps for all inter-hemispheric electrode pairs. (H) Gamma coherence through individual electrodes and (I) electrodes grouped by region. Two-way ANOVA for region: genotype: F(1,315) = 24.86, p < 0.0001; genotype × region interaction: F(4,315) = 0.9112, p = 0.4576; post hoc tests: frontal: p = 0.3285, central: p = 0.0465, parietal: p = 0.0022, temporal: p > 0.9999, occipital: p = 0.1522. NT (black): n = 53, AS (red): n = 12. (PDF 503 kb) [file 13229_2018_214_MOESM2_ESM.pdf]

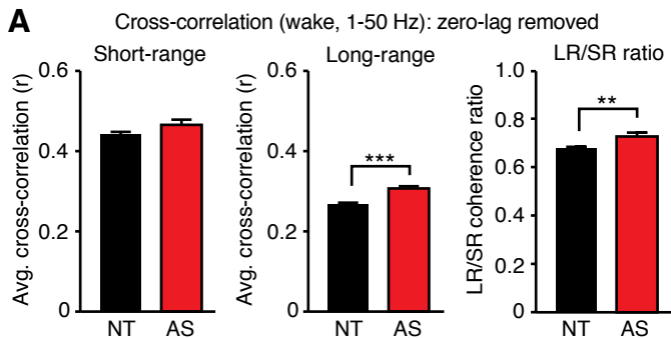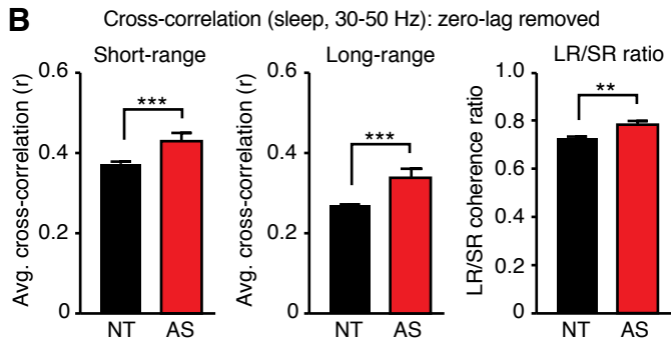

Supplement: Supplementary file 3 — Figure S3. Coherence phenotypes persist with conservative exclusion of volume conduction. (A) Cross-correlation during wakefulness across all frequencies (1–50 Hz). Left panel: short-range electrode pairs (p = 0.0549). Center panel: long-range electrode pairs (p < 0.0001). Right panel: long-range/short-range ratio (p = 0.0027). (B) Cross-correlation during sleep in the gamma band (30–50 Hz). Left panel: short-range (p = 0.0004). Center panel: long-range (p < 0.0001). Right panel: long-range/short-range ratio (p = 0.0016). (PDF 405 kb) [file 13229_2018_214_MOESM3_ESM.pdf]
